# Supplementary figures and images for: Effects of elevated CO2 on predator avoidance behaviour by reef fishes is not altered by experimental test water
Source: PeerJ. 2016 Oct 6;4:e2501. doi: 10.7717/peerj.2501 (PMC5068342; doi:10.7717/peerj.2501)

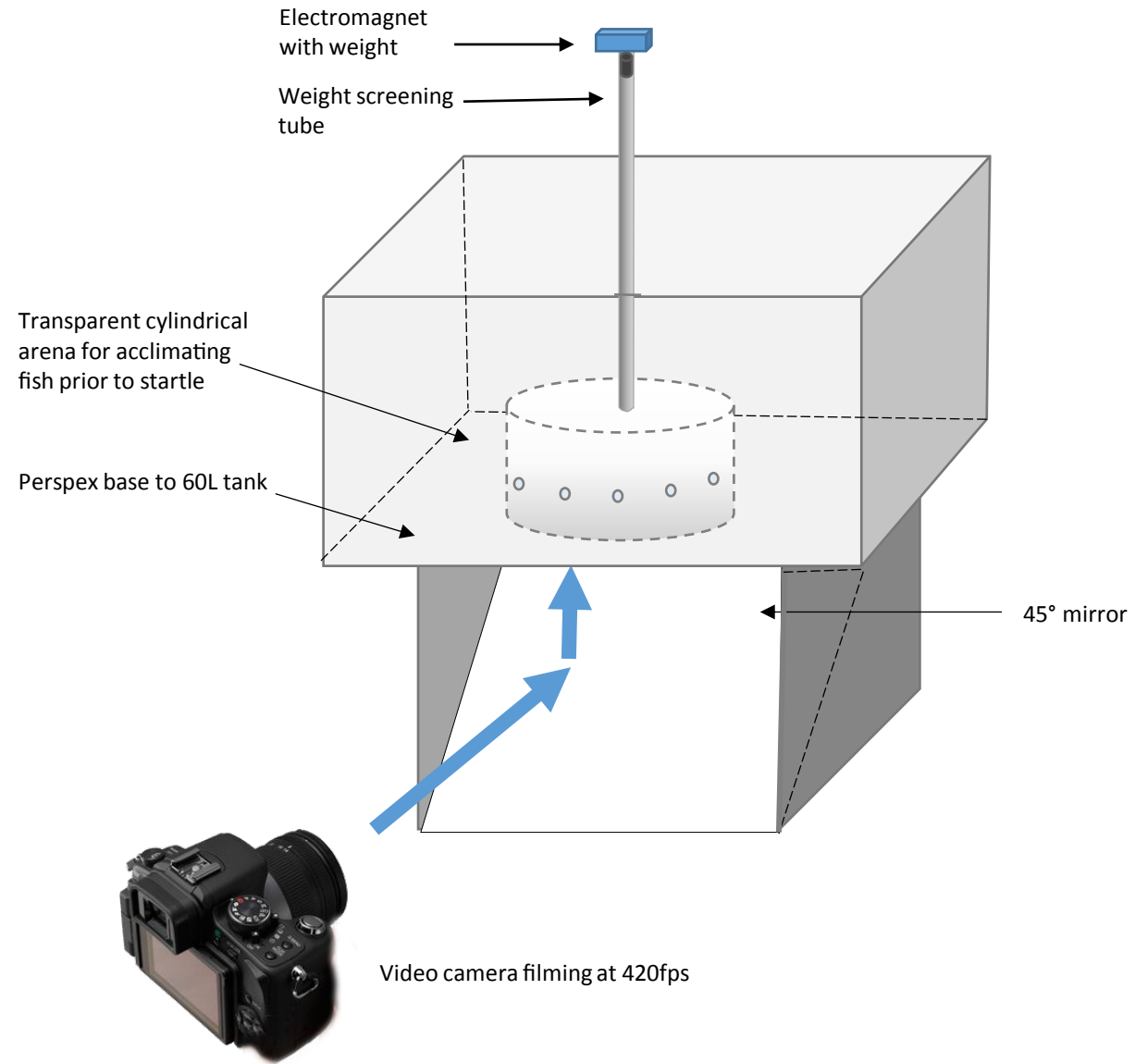

Figure S1. Experimental apparatus for testing escape responses in juvenile reef fish

Supplement: Fig. S1 — Experimental apparatus for testing escape responses in juvenile reef fish [file peerj-04-2501-s001.pdf]
