# Supplementary material for: Effects of elevated CO2 on predator avoidance behaviour by reef fishes is not altered by experimental test water
Source: PeerJ. 2016 Oct 6;4:e2501. doi: 10.7717/peerj.2501 (PMC5068342; doi:10.7717/peerj.2501)
Supplement: Supplemental Information 1 [file peerj-04-2501-s003.docx]

rm(list=ls())

setwd("C:/Users/BLANK/Desktop/")

co2_val <- read.csv("R data Munday et al. 2016 Peer J.csv", header = TRUE)

names(co2_val)

str(co2_val)

co2_val$TANK <-as.factor(co2_val$TANK)

co2_val$DAY <-as.factor(co2_val$DAY)

is.factor(co2_val$TANK)

is.factor(co2_val$DAY)

library(nlme)

newdata <- na.omit(co2_val)

LATENCY.lme = lme(LATENCY ~ TREATMENT + TEST_WATER + DAY + DIST_STIM + TREATMENT : TEST_WATER, random= ~1|TANK, method = "REML", data=newdata)

summary(LATENCY.lme)

anova(LATENCY.lme)

qqnorm(LATENCY.lme$residuals)

qqline(LATENCY.lme$residuals, col="red")

plot(LATENCY.lme) #....or...

E1 <- resid(LATENCY.lme, type = "normalized")

F1 <- fitted(LATENCY.lme)

op <- par(mfrow = c(2, 2), mar = c(4, 4, 3, 2))

MyYlab <- "Residuals"

plot(x = F1, y = E1, xlab = "Fitted values", ylab = MyYlab)

par(mfrow = c(2, 2), mar = c(4, 3, 3, 2))

boxplot(E1 ~ TREATMENT, data = newdata,

main = "TREATMENT", ylab = "Residuals")

boxplot(E1 ~ TEST_WATER, data = newdata,

main = "TEST_WATER", ylab = "Residuals")

boxplot(E1 ~ TANK, data = newdata,

main = "TANK", ylab = "Residuals")

boxplot(E1 ~ DAY, data = newdata,

main = "DAY", ylab = "Residuals")

log.LATENCY<-log(newdata$LATENCY)

log.LATENCY.lme = lme(log.LATENCY ~ TREATMENT + TEST_WATER + DIST_STIM + TREATMENT : TEST_WATER, random= ~1|TANK, method = "REML", data=newdata)

summary(log.LATENCY.lme)

anova(log.LATENCY.lme)

qqnorm(log.LATENCY.lme$residuals)

qqline(log.LATENCY.lme$residuals, col="red")

plot(log.LATENCY.lme) #or...

E1 <- resid(log.LATENCY.lme, type = "normalized")

F1 <- fitted(log.LATENCY.lme)

op <- par(mfrow = c(2, 2), mar = c(4, 4, 3, 2))

MyYlab <- "Residuals"

plot(x = F1, y = E1, xlab = "Fitted values", ylab = MyYlab)

par(mfrow = c(2, 2), mar = c(4, 3, 3, 2))

boxplot(E1 ~ TREATMENT, data = newdata,

main = "TREATMENT", ylab = MyYlab)

boxplot(E1 ~ TEST_WATER, data = newdata,

main = "TEST_WATER", ylab = MyYlab)

boxplot(E1 ~ TANK, data = newdata,

main = "TANK", ylab = MyYlab)

boxplot(E1 ~ DAY, data = newdata,

main = "DAY", ylab = MyYlab)

newdata$dummy = 1

log.LATENCY.lme1 = lme(log.LATENCY ~ TREATMENT + TEST_WATER + DAY + DIST_STIM + TREATMENT : TEST_WATER, random= ~1|TANK, data=newdata, method = "REML")

log.LATENCY.lme0 = lme(log.LATENCY ~ TREATMENT + TEST_WATER + DAY + DIST_STIM + TREATMENT : TEST_WATER, random= ~1|dummy, data=newdata, method = "REML")

anova(log.LATENCY.lme0,log.LATENCY.lme1 )

log.LATENCY.lme1.hetvar = lme(log.LATENCY ~ TREATMENT + TEST_WATER + DAY + DIST_STIM + TREATMENT : TEST_WATER,

random= ~1|TANK, data=newdata, weights = varIdent(form= ~1|TREATMENT), method = "REML")

anova(log.LATENCY.lme1, log.LATENCY.lme1.hetvar)

qqnorm(log.LATENCY.lme1.hetvar$residuals)

qqline(log.LATENCY.lme1.hetvar$residuals, col="red")

E1 <- resid(log.LATENCY.lme1.hetvar, type = "normalized")

F1 <- fitted(log.LATENCY.lme1.hetvar)

par(mfrow = c(2, 2), mar = c(4, 3, 3, 2))

boxplot(E1 ~ TREATMENT, data = newdata,

main = "TREATMENT", ylab = "Residuals")

boxplot(E1 ~ TEST_WATER, data = newdata,

main = "TEST_WATER", ylab = "Residuals")

boxplot(E1 ~ TANK, data = newdata,

main = "TANK", ylab = "Residuals")

boxplot(E1 ~ DAY, data = newdata,

main = "DAY", ylab = "Residuals")

library(MuMIn)

log.LATENCY.lme1.hetvar = lme(log.LATENCY ~ TREATMENT + TEST_WATER + DAY + DIST_STIM + TREATMENT : TEST_WATER, random= ~1|TANK,

data=newdata, weights = varIdent(form= ~1|TREATMENT), method = "ML")

model.set<-dredge(log.LATENCY.lme1.hetvar)

model.set

top.models.p.2<-get.models(model.set,subset=delta<2)

summary(top.models.p.2)

summary(model.avg(top.models.p.2))

top.models.p.2

dredging<-summary(model.avg(get.models(subset(dredge(log.LATENCY.lme1.hetvar,rank="AICc")),delta<2)))

dredging

BEST.log.LATENCYlme = lme(log.LATENCY~ TREATMENT, random= ~1|TANK, data=newdata, weights = varIdent(form= ~1|TREATMENT),

method = "REML")

summary(BEST.log.LATENCYlme)

anova(BEST.log.LATENCYlme)

windows()

qqnorm(BEST.log.LATENCYlme$residuals)

qqline(BEST.log.LATENCYlme$residuals, col="red")

plot(BEST.log.LATENCYlme) #or...

E1 <- resid(BEST.log.LATENCYlme, type = "normalized")

F1 <- fitted(BEST.log.LATENCYlme)

op <- par(mfrow = c(2, 2), mar = c(4, 4, 3, 2))

MyYlab <- "Residuals"

plot(x = F1, y = E1, xlab = "Fitted values", ylab = MyYlab)

par(mfrow = c(2, 2), mar = c(4, 3, 3, 2))

boxplot(E1 ~ TREATMENT, data = newdata,

main = "TREATMENT", ylab = "Residuals")

boxplot(E1 ~ TEST_WATER, data = newdata,

main = "TEST_WATER", ylab = "Residuals")

boxplot(E1 ~ TANK, data = newdata,

main = "TANK", ylab = "Residuals")

boxplot(E1 ~ DAY, data = newdata,

main = "DAY", ylab = "Residuals")

#MAX_SPEED

MAX_SPEED.lme = lme(MAX_SPEED ~ TREATMENT + TEST_WATER + DAY + DIST_STIM + TREATMENT : TEST_WATER, random= ~1|TANK, method = "REML", data=co2_val)

summary(MAX_SPEED.lme)

anova(MAX_SPEED.lme)

qqnorm(MAX_SPEED.lme$residuals)

qqline(MAX_SPEED.lme$residuals, col="red")

plot(MAX_SPEED.lme)

E1 <- resid(MAX_SPEED.lme, type = "normalized")

F1 <- fitted(MAX_SPEED.lme)

op <- par(mfrow = c(2, 2), mar = c(4, 4, 3, 2))

MyYlab <- "Residuals"

plot(x = F1, y = E1, xlab = "Fitted values", ylab = MyYlab)

par(mfrow = c(2, 2), mar = c(4, 3, 3, 2))

boxplot(E1 ~ TREATMENT, data = co2_val,

main = "TREATMENT", ylab = "Residuals")

boxplot(E1 ~ TEST_WATER, data = co2_val,

main = "TEST_WATER", ylab = "Residuals")

boxplot(E1 ~ TANK, data = co2_val,

main = "TANK", ylab = "Residuals")

boxplot(E1 ~ DAY, data = co2_val,

main = "DAY", ylab = "Residuals")

co2_val$dummy = 1

MAX_SPEED.lme1 = lme(MAX_SPEED ~ TREATMENT + TEST_WATER + DAY + DIST_STIM + TREATMENT : TEST_WATER, random= ~1|TANK, data=co2_val, method = "REML")

MAX_SPEED.lme0 = lme(MAX_SPEED ~ TREATMENT + TEST_WATER + DAY + DIST_STIM + TREATMENT : TEST_WATER, random= ~1|dummy, data=co2_val, method = "REML")

anova(MAX_SPEED.lme0,MAX_SPEED.lme1 )

plot(MAX_SPEED.lme1)

qqnorm(MAX_SPEED.lme1$residuals)

qqline(MAX_SPEED.lme1$residuals, col="red")

library(MuMIn)

MAX_SPEED.lme1 = lme(MAX_SPEED ~ TREATMENT + TEST_WATER + DAY + DIST_STIM + TREATMENT : TEST_WATER,

random= ~1|TANK, data=co2_val, method = "ML")

MAX_SPEED.set<-dredge(MAX_SPEED.lme1)

MAX_SPEED.set

top.models.p.2<-get.models(model.set,subset=delta<2)

summary(top.models.p.2)

summary(model.avg(top.models.p.2))

BEST.MAX_SPEEDlme = lme(MAX_SPEED ~ TREATMENT, random= ~1|TANK, data=co2_val, method = "REML")

summary(BEST.MAX_SPEEDlme)

anova(BEST.MAX_SPEEDlme)

qqnorm(BEST.MAX_SPEEDlme$residuals)

qqline(BEST.MAX_SPEEDlme$residuals, col="red")

plot(BEST.MAX_SPEEDlme) #or...

E1 <- resid(BEST.MAX_SPEEDlme, type = "normalized")

F1 <- fitted(BEST.MAX_SPEEDlme)

op <- par(mfrow = c(2, 2), mar = c(4, 4, 3, 2))

MyYlab <- "Residuals"

plot(x = F1, y = E1, xlab = "Fitted values", ylab = MyYlab)

par(mfrow = c(2, 2), mar = c(4, 3, 3, 2))

boxplot(E1 ~ TREATMENT, data = co2_val,

main = "TREATMENT", ylab = "Residuals")

boxplot(E1 ~ TEST_WATER, data = co2_val,

main = "TEST_WATER", ylab = "Residuals")

boxplot(E1 ~ TANK, data = co2_val,

main = "TANK", ylab = "Residuals")

boxplot(E1 ~ DAY, data = co2_val,

main = "DAY", ylab = "Residuals")

#SPEED

SPEED.lme = lme(SPEED ~ TREATMENT + TEST_WATER + DAY + DIST_STIM + TREATMENT : TEST_WATER, random= ~1|TANK, method = "REML", data=co2_val)

summary(SPEED.lme)

anova(SPEED.lme)

qqnorm(SPEED.lme$residuals)

qqline(SPEED.lme$residuals, col="red")

plot(SPEED.lme)

E1 <- resid(SPEED.lme, type = "normalized")

F1 <- fitted(SPEED.lme)

op <- par(mfrow = c(2, 2), mar = c(4, 4, 3, 2))

MyYlab <- "Residuals"

plot(x = F1, y = E1, xlab = "Fitted values", ylab = MyYlab)

par(mfrow = c(2, 2), mar = c(4, 3, 3, 2))

boxplot(E1 ~ TREATMENT, data = co2_val,

main = "TREATMENT", ylab = "Residuals")

boxplot(E1 ~ TEST_WATER, data = co2_val,

main = "TEST_WATER", ylab = "Residuals")

boxplot(E1 ~ TANK, data = co2_val,

main = "TANK", ylab = "Residuals")

boxplot(E1 ~ DAY, data = co2_val,

main = "DAY", ylab = "Residuals")

co2_val$dummy = 1

SPEED.lme1 = lme(SPEED ~ TREATMENT + TEST_WATER + DAY + DIST_STIM + TREATMENT : TEST_WATER, random= ~1|TANK, data=co2_val, method = "REML")

SPEED.lme0 = lme(SPEED ~ TREATMENT + TEST_WATER + DAY + DIST_STIM + TREATMENT : TEST_WATER, random= ~1|dummy, data=co2_val, method = "REML")

anova(SPEED.lme0,SPEED.lme1 )

library(MuMIn)

SPEED.lme1 = lme(SPEED ~ TREATMENT + TEST_WATER + DAY + DIST_STIM + TREATMENT : TEST_WATER,

random= ~1|TANK, data=co2_val, method = "ML")

SPEED.set<-dredge(SPEED.lme1)

SPEED.set

top.models.p.2<-get.models(model.set,subset=delta<2)

summary(top.models.p.2)

summary(model.avg(top.models.p.2))

BEST.SPEEDlme1 = lme(SPEED ~ DAY + TREATMENT, random= ~1|TANK, data=co2_val, method = "REML")

summary(BEST.SPEEDlme1)

anova(BEST.SPEEDlme1)

qqnorm(BEST.SPEEDlme1$residuals)

qqline(BEST.SPEEDlme1$residuals, col="red")

plot(BEST.SPEEDlme1)

E1 <- resid(BEST.SPEEDlme1, type = "normalized")

F1 <- fitted(BEST.SPEEDlme1)

op <- par(mfrow = c(2, 2), mar = c(4, 4, 3, 2))

MyYlab <- "Residuals"

plot(x = F1, y = E1, xlab = "Fitted values", ylab = MyYlab)

par(mfrow = c(2, 2), mar = c(4, 3, 3, 2))

boxplot(E1 ~ TREATMENT, data = co2_val,

main = "TREATMENT", ylab = "Residuals")

boxplot(E1 ~ TEST_WATER, data = co2_val,

main = "TEST_WATER", ylab = "Residuals")

boxplot(E1 ~ TANK, data = co2_val,

main = "TANK", ylab = "Residuals")

boxplot(E1 ~ DAY, data = co2_val,

main = "DAY", ylab = "Residuals")

#DISTANCE

DISTANCE.lme = lme(DISTANCE ~ TREATMENT + TEST_WATER + DAY + DIST_STIM + TREATMENT : TEST_WATER, random= ~1|TANK, method = "REML", data=co2_val)

summary(DISTANCE.lme)

anova(DISTANCE.lme)

qqnorm(DISTANCE.lme$residuals)

qqline(DISTANCE.lme$residuals, col="red")

plot(DISTANCE.lme)

E1 <- resid(DISTANCE.lme, type = "normalized")

F1 <- fitted(DISTANCE.lme)

op <- par(mfrow = c(2, 2), mar = c(4, 4, 3, 2))

MyYlab <- "Residuals"

plot(x = F1, y = E1, xlab = "Fitted values", ylab = MyYlab)

par(mfrow = c(2, 2), mar = c(4, 3, 3, 2))

boxplot(E1 ~ TREATMENT, data = co2_val,

main = "TREATMENT", ylab = "Residuals")

boxplot(E1 ~ TEST_WATER, data = co2_val,

main = "TEST_WATER", ylab = "Residuals")

boxplot(E1 ~ TANK, data = co2_val,

main = "TANK", ylab = "Residuals")

boxplot(E1 ~ DAY, data = co2_val,

main = "DAY", ylab = "Residuals")

co2_val$dummy = 1

DISTANCE.lme1 = lme(DISTANCE ~ TREATMENT + TEST_WATER + DAY + DIST_STIM + TREATMENT : TEST_WATER, random= ~1|TANK, data=co2_val, method = "REML")

DISTANCE.lme0 = lme(DISTANCE ~ TREATMENT + TEST_WATER + DAY + DIST_STIM + TREATMENT : TEST_WATER, random= ~1|dummy, data=co2_val, method = "REML")

anova(DISTANCE.lme0,DISTANCE.lme1)

plot(DISTANCE.lme1)

qqnorm(DISTANCE.lme1$residuals)

qqline(DISTANCE.lme1$residuals, col="red")

library(MuMIn)

DISTANCE.lme1 = lme(DISTANCE ~ TREATMENT + TEST_WATER + DAY + DIST_STIM + TREATMENT : TEST_WATER,

random= ~1|TANK, data=co2_val, method = "ML")

model.set<-dredge(DISTANCE.lme1)

model.set

top.models.p.2<-get.models(model.set,subset=delta<2)

summary(top.models.p.2)

summary(model.avg(top.models.p.2))

BEST.DISTANCElme = lme(DISTANCE ~ TREATMENT + DAY, random= ~1|TANK, data=co2_val, method = "REML")

summary(BEST.DISTANCElme)

anova(BEST.DISTANCElme)

qqnorm(BEST.DISTANCElme$residuals)

qqline(BEST.DISTANCElme$residuals, col="red")

plot(BEST.DISTANCElme) #or...

E1 <- resid(BEST.DISTANCElme, type = "normalized")

F1 <- fitted(BEST.DISTANCElme)

op <- par(mfrow = c(2, 2), mar = c(4, 4, 3, 2))

MyYlab <- "Residuals"

plot(x = F1, y = E1, xlab = "Fitted values", ylab = MyYlab)

par(mfrow = c(2, 2), mar = c(4, 3, 3, 2))

boxplot(E1 ~ TREATMENT, data = co2_val,

main = "TREATMENT", ylab = "Residuals")

boxplot(E1 ~ TEST_WATER, data = co2_val,

main = "TEST_WATER", ylab = "Residuals")

boxplot(E1 ~ TANK, data = co2_val,

main = "TANK", ylab = "Residuals")

boxplot(E1 ~ DAY, data = co2_val,

main = "DAY", ylab = "Residuals")

###########END#######################################################
